# Clinical Outcomes of Revascularization Strategies for Patients With MVD/LMCA Disease: A Systematic Review and Network Meta-Analysis

**DOI:** 10.1097/MD.0000000000001745

**Published:** 2015-10-23

**Authors:** Meng-Kan Fan, Ya-Min Su, Xing-Xing Cai, Zhou-Shan Gu, Hai-Hua Geng, Hai-Yan Pan, Jian-Hua Zhu, Min Pan

**Affiliations:** From the Department of Cardiology, Affiliated Hospital of Nantong University, Nantong, People's Republic of China.

## Abstract

Hybrid coronary revascularization (HCR), a new minimally invasive procedure for patients requiring revascularization for multivessel coronary lesions, combines coronary artery bypass grafting (CABG) for left anterior descending (LAD) lesions and percutaneous coronary intervention (PCI) for non-LAD coronary lesions. However, available data related to outcomes comparing the 3 revascularization therapies is limited to small studies.

We conducted a search in MEDLINE, EMBASE, and the Cochrane Library of Controlled Trials up to December 31, 2014, without language restriction. A total of 16 randomized trials (n=4858 patients) comparing HCR versus PCI or off-pump CABG (OPCAB) were included in this meta-analysis. The primary outcomes were major adverse cardiac and cerebrovascular events (MACCE), all-cause death, myocardial infarction (MI), cerebrovascular events (CVE), and target vessel revascularization (TVR). Odds ratios (OR) and 95% confidence intervals (CI) were calculated using random-effect and fixed-effect models. Ranking probabilities were used to calculate a summary numerical value: the surface under the cumulative ranking (SUCRA) curve.

No significant differences were seen between the HCR and PCI in short term (in hospital and 30 days) with regard to MACCE (odds ratio [OR] = 0.51, 95% confidence interval [CI] 0.00–2.35), all-cause death (OR = 2.09, 95% CI 0.34–7.66), MI (OR = 1.02, 95% CI 0.19–2.95), CVE (OR = 4.45, 95% CI 0.39–19.16), and TVR (OR = 6.99, 95% CI 0.17–39.39). However, OPCAB had lower MACCE than HCR (OR = 0.19, 95% CI 0.00–0.95). In midterm (1 year and 3 year), in comparison with HCR, PCI had higher all-cause death (OR = 5.66, 95% CI 0.00–13.88) and CVE (OR = 4.40, 95% CI 0.01–5.68), and lower MI (OR = 0.51, 95% CI 0.00–2.86), TVR (OR = 0.53, 95% CI 0.05–2.26), and thus the MACCE (OR = 0.51, 95% CI 0.00–2.35). Off-pump CABG presented a better outcome than HCR with significant lower MACCE (OR = 0.17, 95% CI 0.01–0.68). Surface under the cumulative ranking probabilities showed that HCR may be the superior strategy for MVD and LMCA disease when regarded to MACCE (SUCRA = 0.84), MI (SUCRA = 0.76) in short term, and regarded to MACCE (SUCRA = 0.99), MI (SUCRA = 0.94), and CVE (SUCRA = 0.92) in midterm.

Hybrid coronary revascularization seemed to be a feasible and acceptable option for treatment of LMCA disease and MVD. More powerful evidences are required to precisely evaluate risks and benefits of the 3 therapies for patients who have different clinical characteristics.

## INTRODUCTION

Coronary artery disease (CAD) has been proved to be one of the major threats to health. More than 7 million deaths attributed to CAD each year worldwide.^1^ Both coronary artery bypass grafting (CABG) and percutaneous coronary intervention (PCI) offer certain benefits for patients with multivessel coronary artery disease (MVD) or left main coronary artery (LMCA) disease.

Off-pump coronary artery bypass grafting (OPCAB) has been considered the optimum revascularization treatment for patients with LMCA disease and/or 3-vessel disease. In the past 2 decades, PCI has emerged as a possible alternative for patients with complex coronary disease because of the improved stent design, procedural technique, and adjunctive medical therapy.^2,3^ Hybrid coronary revascularization (HCR), which combines CABG for left anterior descending (LAD) lesions and PCI for non-LAD coronary lesions,^4,5^ is a new minimally invasive procedure for patients requiring revascularization to deal with multivessel coronary lesions.

Hybrid coronary revascularization seems to be a feasible therapy strategy for MVD or LMCA disease. However, there is little comparison of outcomes between PCI and HCR. The purpose of this study was thus to perform a systematic review and network meta-analysis in order to compare clinical outcomes of the 3 revascularizations.

## METHODS

### Strategy for Literature Search

MEDLINE, EMBASE, and the Cochrane Library were searched with following terms to achieve eligible clinical evidence with following terms: “hybrid,” “hybrid revascularization,” “coronary artery bypass,” “OPCAB,” “minimally invasive direct coronary artery bypass,” “PCI,” “stents,” and “drug-eluting.” Furthermore, the reference lists from retrieved articles were checked to search for further relevant studies. Two investigators (P.M. and F.M.K.) searched for the literature independently, with conflicts resolved by discussion. All searches for the literature were executed on December 31, 2014. This study did not involve human subjects, so informed consent was not required. In addition, no approval was required from an institutional review board.

### Study Selection

Studies were included if they met all criteria as follows: (i) adult patients diagnosed with MVD or LMCA disease by coronary angiography; (ii) details of outcomes of any two of OPCAB/PCI/HCR were described.(iii) sufficient original data were getable from the full text or by contacting with authors for analysis.

### Outcomes and Data Extraction and Quality Appraisal

The primary clinical outcomes of interest were major adverse cardiac and cerebrovascular events (MACCE), all-cause death, myocardial infarction (MI), cerebrovascular events (CVE), and target vessel revascularization (TVR). Major adverse cardiac and cerebrovascular events included death of any cause, nonfatal MI, CVE, and repeat revascularization by percutaneous intervention or surgery. Time points for analysis were in-hospital, 30 days, 12 months, 3 years, and 5 years. We appraised quality by using the Newcastle–Ottawa scale for cohort studies,^6^ and by using Cochrane Collaboration's risk of bias assessment tool for RCTs.^7^ Included studies were extracted and apprised by 2 investigators (P.M. and F.M.K.) independently. After disagreements resolved by consensus, they reviewed all data to ensure accuracy before analysis.

### Analysis

All continuous variables are expressed as the mean ± standard deviation. Network meta-analysis was performed within a Bayesian framework computing odds ratios (95% confidence interval) with a random-effect model, sampling posterior probabilities by means of Markov chain Monte Carlo (MCMC) methods with Gibbs sampling from 50,000 iterations obtained after a 10,000-iteration training phase.^8,9^ One MCMC chain was used to assess convergence using Brooks–Gelman–Rubin plots. Sensitivity was assessed by the deviance information criterion (DIC), which yielded by computing difference from a random-effect and a fixed-effect model. The potential for inconsistency was assessed by comparing direct and indirect estimates. Ranking probabilities were used to calculate a summary numerical value: *t* the surface under the cumulative ranking (SUCRA) curve.^10^ Stata12 and WinBUGS14 were used in all statistical analyses.^8^

## RESULTS

### Eligible Studies

**FIGURE 1:**
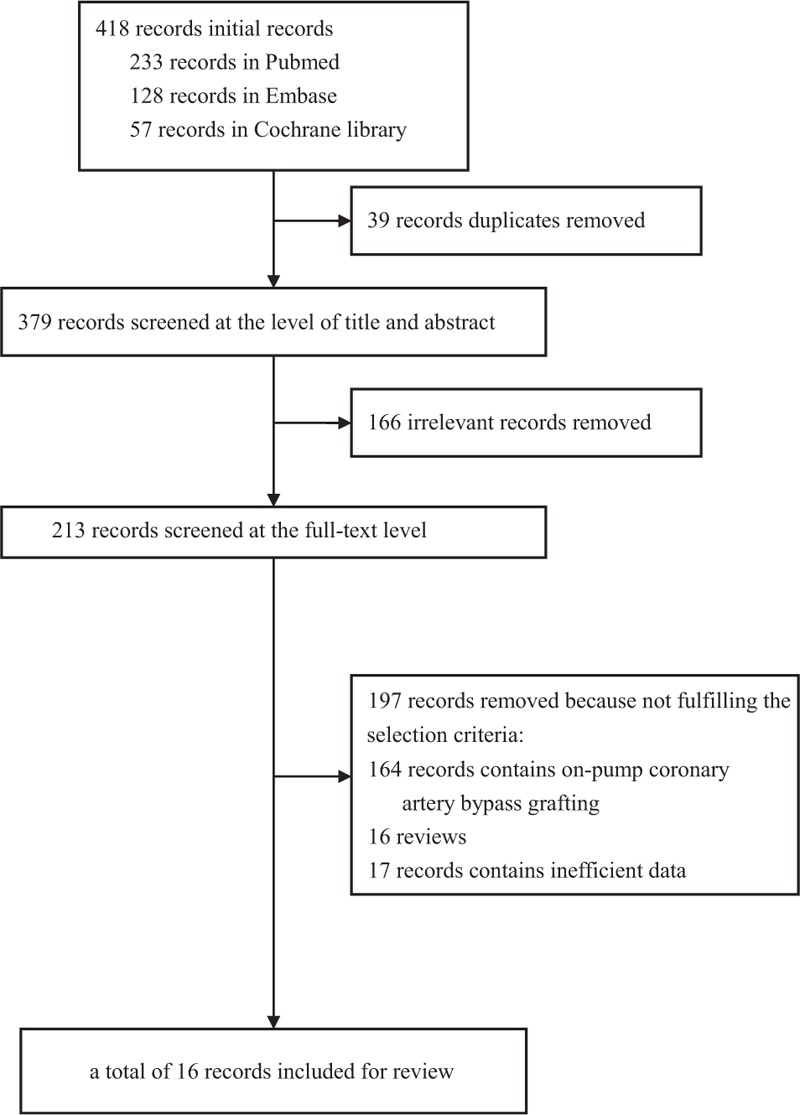
Flowchart of the inclusion process of the studies for network meta-analysis.

The inclusion process of the studies for network meta-analysis is shown in Figure 1. A total of 418 citations were yielded by our searching strategy from multiple databases, finally 16 studies ^4,11–25^ were eligible for our systematic review. These trials included a total of 4858 patients, according to the following quantitative synthesis: 9 studies ^11–18,22,23^ compared PCI with OPCAB, 7 studies ^4,19–22,24,25^ compared HCR with OPCAB, and all studies were observational cohorts (OC). A total of 1723 patients from 9 studies ^11–17,23^ underwent PCI with DES, 2643 patients from all 16 studies ^4,11–25^ underwent OPCAB, and 492 patients from 7 studies^4,19–22,24,25^ underwent HCR. Three studies ^12,18,19^ underwent revascularization for LMCA disease, 12 studies ^4,11,13–15,17,20–25^ underwent MVD revascularization, whereas the remaining 1 study ^16^ did not supply detailed data of subgroup of LMCA disease or MVD.

**FIGURE 2:**
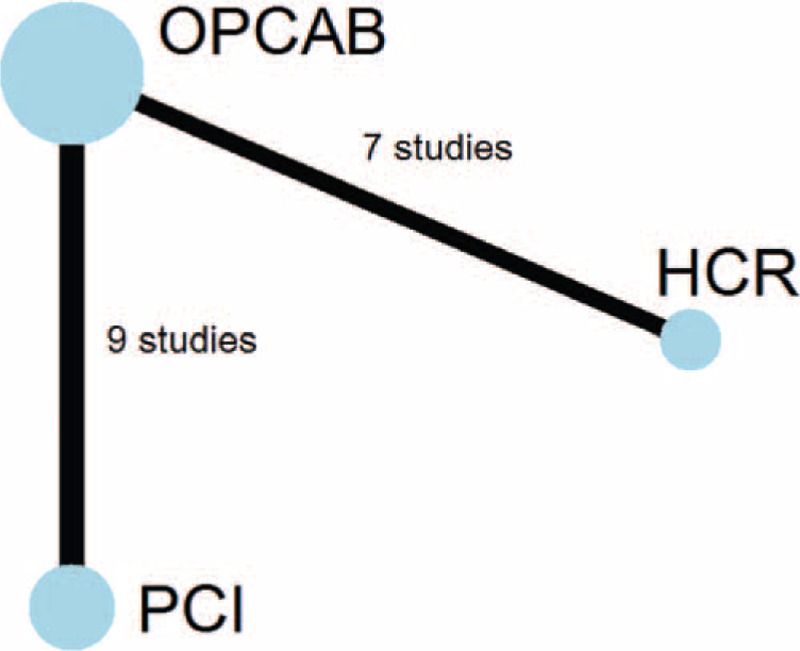
Network of comparisons included in analyses. Circle size reflects sample size, whereas line width is proportional to the number of comparisons. HCR = hybrid coronary revascularization, OPCAB = off-pump coronary artery bypass grafting, PCI = percutaneous coronary intervention.

We divided Sata and his colleague's report ^15^ into 2 studies, Sata 2009 and Sata 2009a, because outcomes of OPCAB versus DES were in 2 different cohorts grouped by the age. We combined data of outcomes of in-hospital and 30 days as short-term outcomes, and 1 year and 3 year as midterm outcomes for following analysis. Because few study reported outcomes longer than 3 year, our research only dealt with short-term and midterm outcomes. Characteristic of studies in our network meta-analysis were summarized in Table 1. All of studies enrolled were OCs. According to Table 2, these studies met most of the Newcastle–Ottawa scale criteria. The evidence network is shown in Figure 2. The clinical characteristics of patients in the included studies are depicted in Table 3. Table 4 showed the events of the primary clinical outcomes of each trial.

**TABLE 1:**
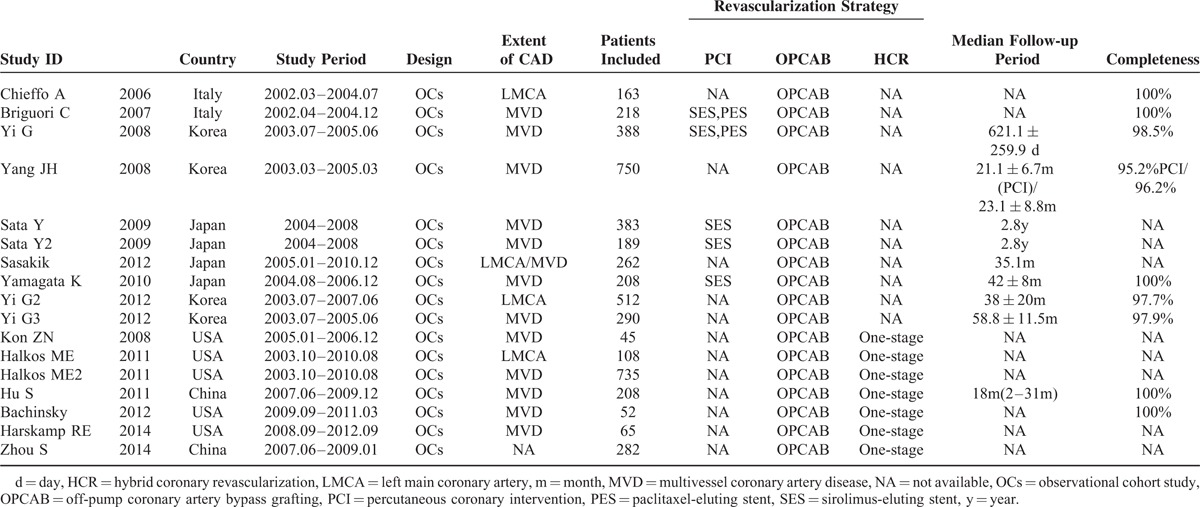
Characteristics of Included Studies

**TABLE 2:**
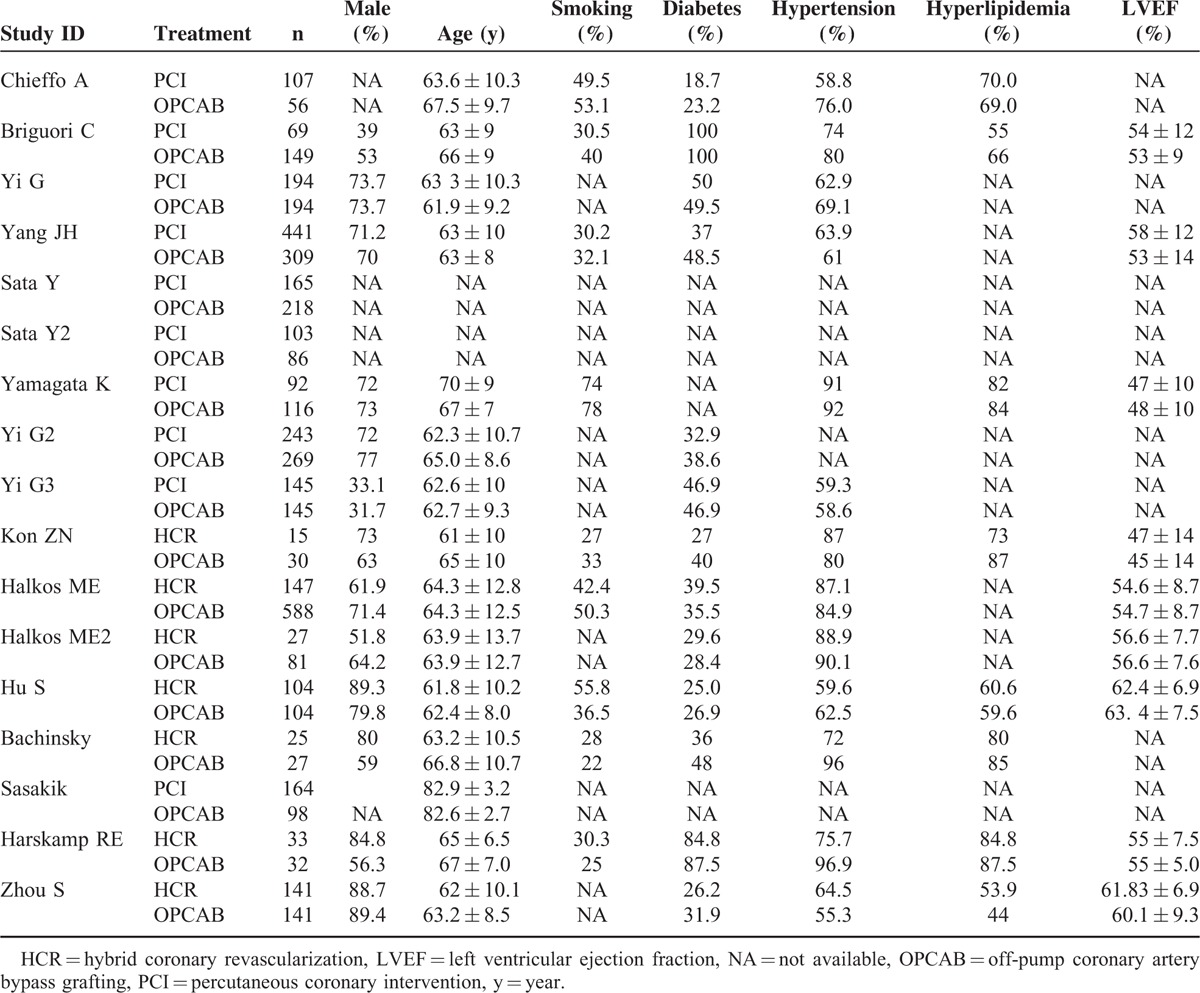
Baseline Characteristic of Patients in the Included Studies

**TABLE 3:**
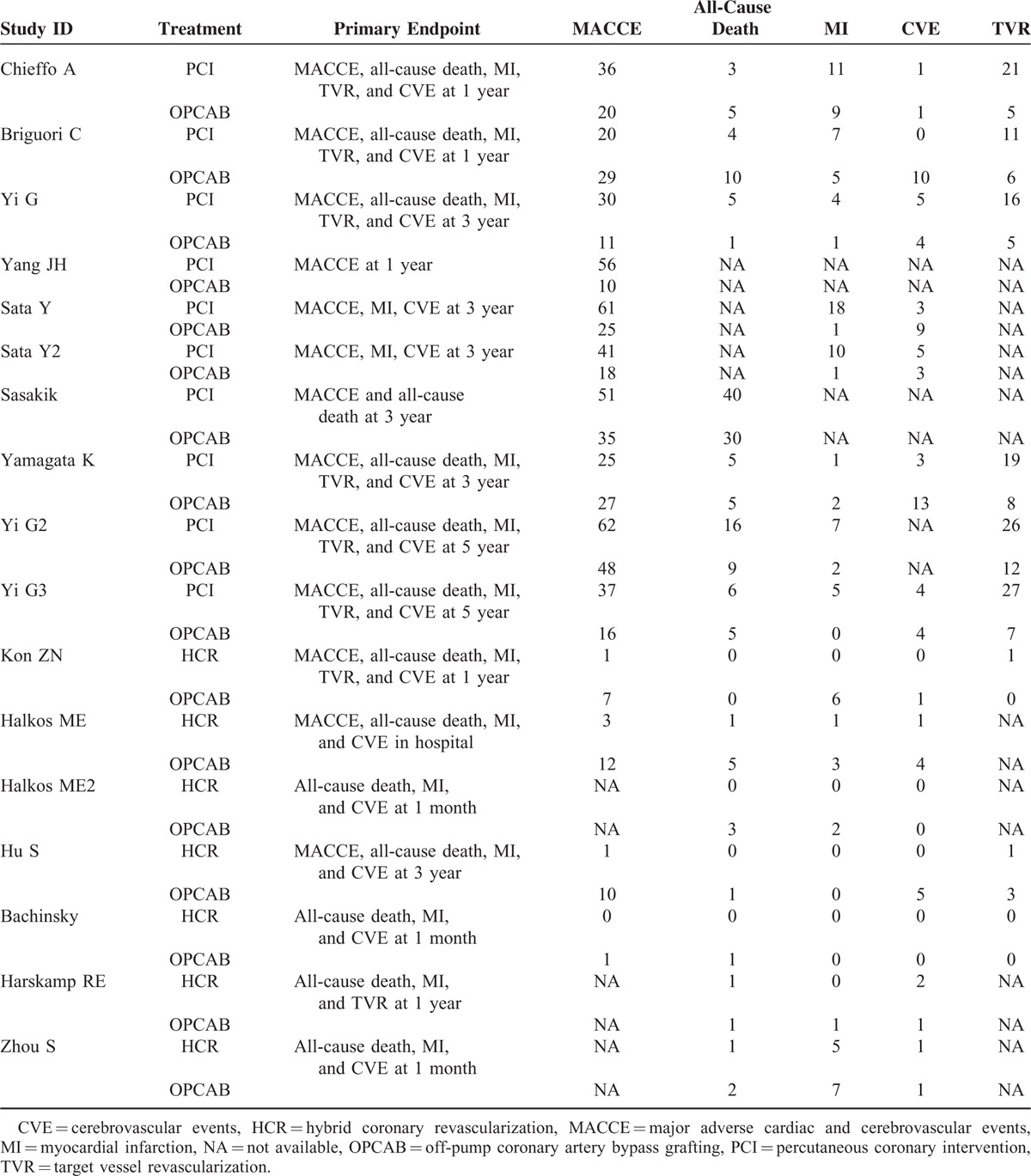
Events of the Primary Clinical Outcomes of Each Included Studies

**TABLE 4:**
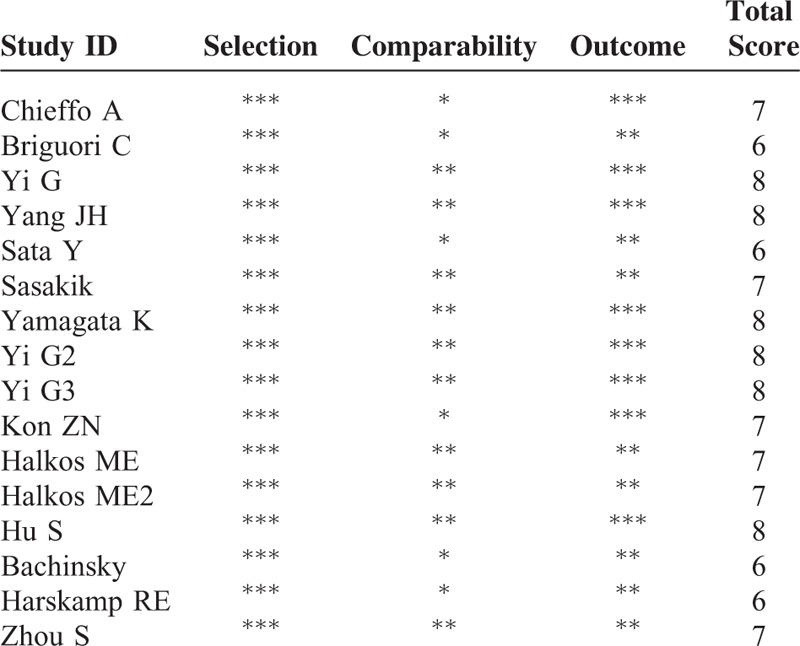
Quality Appraisal Based on the Newcastle–Ottawa Scale

## CLINICAL OUTCOMES

### Short-Term Clinical Outcomes

**FIGURE 3:**
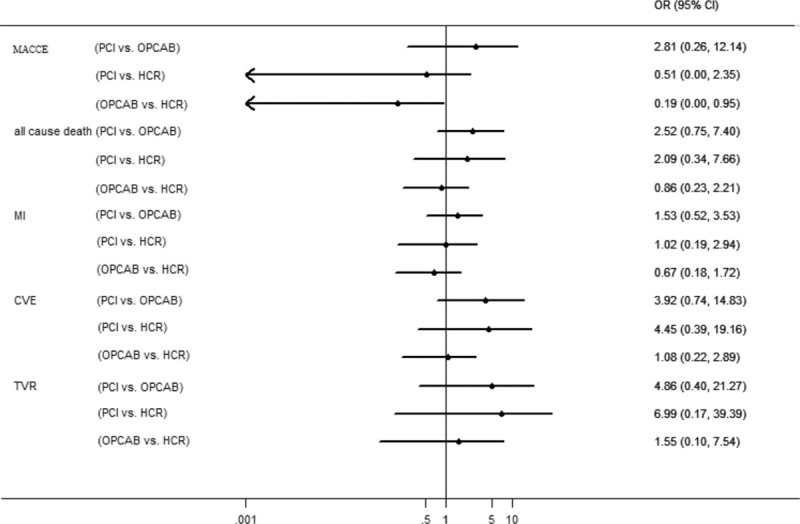
Estimated odds ratios (95% confidence interval) for short-term outcomes of the 3 revascularization therapies.

A total of 13 studies reported short-term clinical outcomes for network-meta analysis, in which 11 studies^4,11–14,17–20,24,25^ reported incidence of MACCE, 13 studies^1,11–13,15,17–25^ reported incidence of death, 11 studies^4,10–12,16–19,21,23,24^ reported incidence of MI, 11 studies^4,11–13,17–20,22,24,25^ reported incidence of CVE, and 8 studies^4,11–13,17,18,20,24^ reported incidence of TVR. As shown in Figure 3, no statistical differences were detected when indirectly comparing MACCE (OR = 0.51, 95% CI 0.00–2.35) and MI (OR = 1.02, 95% CI 0.19–2.95) of PCI and HCR at short term. All-cause death (OR = 2.09, 95% CI 0.34–7.66), CVE (OR = 4.45, 95% CI 0.39–19.16), and TVR (OR = 6.99, 95% CI 0.17–39.39) despite a higher trend in the PCI group did not differ considerably in the 2 groups. Off-pump CABG showed better short-term outcome than PCI without statistical difference. However, OPCAB had lower MACCE than HCR (OR = 0.19, 95% CI 0.00–0.95).

### Midterm Clinical Outcomes

**FIGURE 4:**
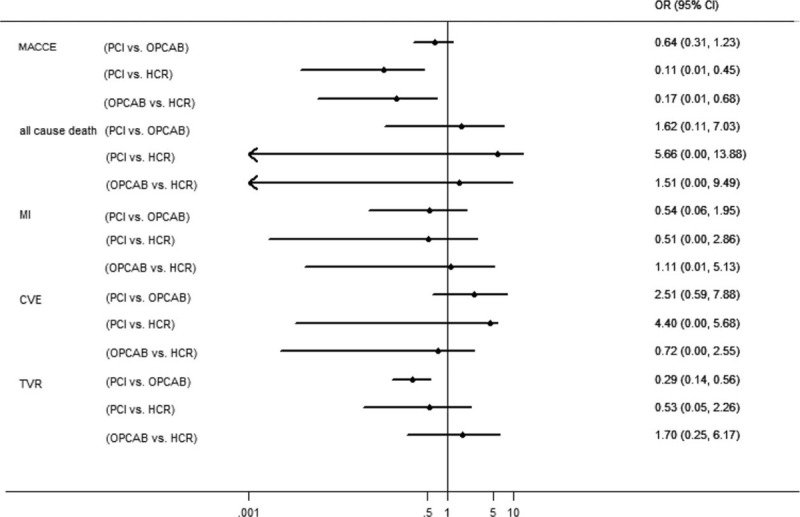
Estimated odds ratios (95% confidence interval) for midterm outcomes of the 3 revascularization therapies.

A total of 10 studies have details of data of midterm clinical outcomes for network-meta analysis. Nine studies^4,11–15,17,23,24^ reported incidence of MACCE, 7 studies^4,11–13,17,21,24^ reported incidence of all-cause death, 8 studies^4,11–13,15,17,21,24^ reported incidence of MI, 7 studies^4,11–13,15,17,24^ reported incidence of CVE, and 7 studies^4,11–13,17,21,24^ reported incidence of TVR. The midterm clinical outcomes comparing the 3 revascularization therapies are presented in Figure 4. In comparison with HCR, PCI had higher all-cause death (OR = 5.66, 95% CI 0.00–13.88) and CVE (OR = 4.40, 95% CI 0.01–5.68), and lower MI (OR = 0.51, 95% CI 0.00–2.86), TVR (OR = 0.53, 95% CI 0.05–2.26), and thus the MACCE (OR = 0.51, 95% CI 0.00–2.35) without statistical significance.

### Ranking Probabilities Analysis

According to Table 5 and Table 6, the surface under the cumulative ranking curve (SUCRA) ordering from the best to the worst, in short term, HCR was ranked first for the lower rate of the primary outcomes-MACCE (SUCRA = 0.86), MI(SUCRA = 0.76). PCI was ranked first for all-cause death (SUCRA = 0.62), CVE (SUCRA = 0.89) and TVR (SUCRA = 0.84). In midterm, HCR was ranked first for MACCE (SUCRA = 0.99), MI (SUCRA = 0.95), and CVE (SUCRA = 0.92). For all-cause death PCI (SUCRA = 0.48), OPCAB (SUCRA = 0.47), and HCR (SUCRA = 0.49) had parallel ranking.

**TABLE 5:**
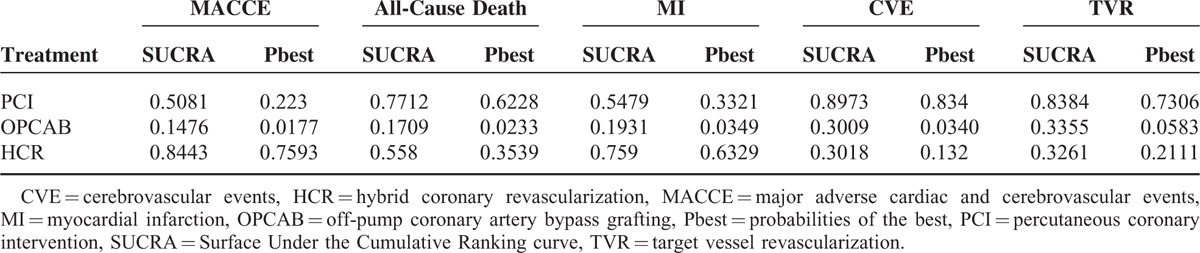
SUCRA Probabilities of the 3 Therapies in Short Term

**TABLE 6:**
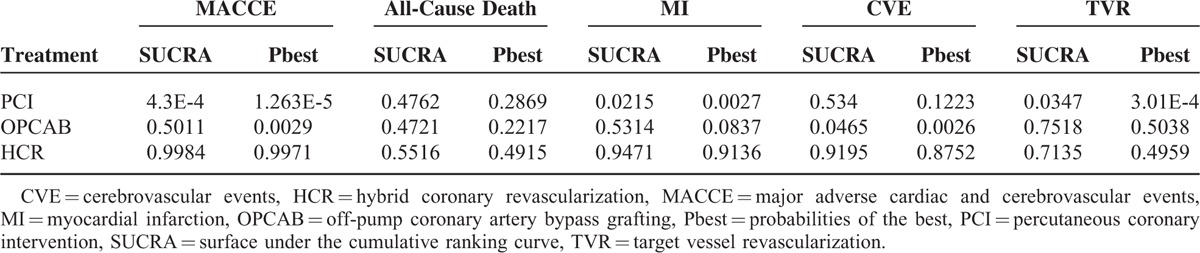
SUCRA Probabilities of the 3 Therapies in Midterm

### Sensitivity and Inconsistence Analysis

In light of the similar results yielded by random-effect and fixed-effect Bayesian models, sensitivity of network-meta was satisfactory. By contrasting results of meta-analysis (based on direct evidence) with network meta-analysis (based on indirect evidence), inconsistency of our network meta-analysis is acceptable. However, inconsistency in network meta-analysis of MACCE at short-term and midterm and TVR at midterm couldn’t be denied.

## DISCUSSION

By taking all results of present network meta-analysis, HCR may have a trend of lower all-cause mortality, CVE and TVR than PCI, whereas it has almost equivalent clinical outcomes compared with OPCAB. In our study, inconsistencies in subsets of MACCE at short-term and midterm and TVR at midterm were detected obviously. Thus the results of such subsets are less persuasive and need to be explained cautiously.

2014 ESC/EACTS Guidelines on Myocardial Revascularization ^26^ demonstrate that CABG is still the superior treatment for severe CAD including proximal LAD, high SYNTAX score of LMCA disease, or MVD. With the progress of technology, OPCAB and minimally invasive CABG through small anterior thoracotomy incisions, which were always being concerned, have significantly developed. However, the limitation of minimally CABG is the difficulty in accessing the posterior and lateral aspects of the heart complete revascularization in many patients with concomitant right coronary artery (RCA) and/or left circumflex (LCX) disease in patients with MVD. Besides, the benefits of CABG surgery remain limited by restnosis of vein graft which could not be neglected.^27^ By contrast, PCI has more complete revascularization for patients who seems like benefit from it.^28^ Recent advances in PCI, especially with usage of new generation drug-eluting stents, have largely expanded the adaptively in revascularization for MVD or LMCA disease.^29^

Hybrid coronary revascularization combines respective advantages of OPCAB and PCI, which use left-internal mammary grafts (LIMA) to LAD and PCI for non-LAD lesions through lateral thoracotomy or by thoracoscope. Several evidences showed that HCR may result in faster recovery, fewer complications compared with CABG in a general population of patients with MVD.^20,25,30,31^

By taking results of present network meta-analysis of PCI, OPCAB, and HCR, the short-term and midterm outcomes of all-cause mortality were similar between any of 2 groups. This result was consistent with the previous meta-analysis.^32,33^ However, HCR has a trend of lower mortality both in short-term and midterm.

The clinical outcomes for MI were also not different in short-term and midterm. Inconsistence analysis did not show significant difference. The same result was drawn by a meta-analysis of Jaffery et al.^34^ They found that overall mortality and myocardial infarction rates were similar in stenting versus MIDCAB but surgery was associated with significantly lower rates of revascularization in comparison to bare metal stenting. In our study, almost all of cohorts used DES for PCI, which maybe a potential reason of less superiority of OPCAB.

There were no statistical differences in the network meta-analyses of CVE at short term and midterm. The studies definitely described data of CVE or stroke were enrolled for analysis. Our analysis is consistent with a meta-analysis of Edelman et al, which showed the rates of stroke between OPCAB and PCI with BMS or DES was similar.^33^

Although the results of cumulative TVR in short term turned into no significant difference between any of 2 groups, we noticed that most of number of events in each study was zero, which may be attributable to efficient pre- and postoperative antiplatelet therapy or protection for graft from distention and trauma.^27^

The ranking probabilities analysis has shown potential benefits of HCR for clinical outcomes. Not only the same results were reported by Phan et al,^32^ but also shorter hospital staying durations than OPCAB. However, HCR have limitations such as operation complications, financial cost, and learning curve, which should be concerned for individual therapy.

This is the first network meta-analysis for revascularization therapies. There were several limitations. First, all the studies enrolled in our research are OCs, in which a majority of trails’ strategies of revascularization selected by doctors or patients may influence the results. Second, not only sum of trials comparing OPCAB versus HCR was less than PCI versus OPCAB, but also the sample size and the number of events were smaller, which may lead to inconsistency of the results. Third, although we combined as much data as possible for network meta-analysis, there was no sufficient data for evaluating outcomes > 3 years for evaluating long-term prognosis. Besides, if more raw data and more high-quality trails were available, more subgroup analyses divided by different characteristics such as gender, age, race, type of CAD, and risk factors could have been done to assess the benefits or risks of therapy selection strategy for individuals. Therefore, more RCTs and original data of trails are required for further network meta-analysis in order to obtain more precise efficient results.

In conclusion, HCR may have almost equivalent short-term outcomes comparing with PCI and OPCAB, whereas it has potential better midterm outcomes. HCR seemed to be a feasible and acceptable option for treatment of LMCA disease and MVD. However, all available studies for this meta-analysis were OCs, so in order to acquire more persuasive indication for selection of revascularization strategies, more powerful evidences are required to precisely evaluate risks and benefits of the 3 therapies for patients who have different clinical characteristics.
